# Social determinants of child abuse: evidence of factors associated with maternal abuse from the Egypt demographic and health survey

**DOI:** 10.5249/jivr.v8i1.630

**Published:** 2016-01

**Authors:** Diddy Antai, Patrick Braithwaite, George Clerk.

**Affiliations:** ^*a*^City University London, School of Health Sciences, Centre for Public Health Research, London, United Kingdom.; ^*b*^Division of Global Health & Inequalities, The Angels Trust - Nigeria, Abuja, Nigeria.; ^*c*^Department of Social and Historical Studies, University of Westminster, London, W1B 2UW, UK.

**Keywords:** Child abuse, Maltreatment, Social determinants, Egypt

## Abstract

**Background:**

Child abuse or maltreatment is a significant global public health problem of unknown global prevalence. About 40 million children aged 0 - 14 years require health and social care globally. The prevalence, determinants, and trends of national or global rates of child abuse and maltreatment are largely unknown.

**Methods:**

Data for this retrospective cross-sectional study were derived from the 2005 Egyptian Demographic and Health Survey (2005 EDHS), and included 19474 women aged 15-49 years. Multivariate logistic regression analyses by stepwise regression, backward method were used to determine the independent contribution of the possible social determinants of child abuse, with the direction and magnitude of associations expressed as odds ratios (OR) and their 95% confident interval levels (95% CI).

**Results:**

Identified determinants of child abuse included exposure to intimate partner violence (IPV), justifying wife beating, exposure to generational IPV, and such factors as younger age of the women, male sex, partners’ lower education, poverty, residence in urban areas, younger children, and residence in households with 3 - 5 children.

**Conclusions:**

Experience of IPV, mothers’ justification of wife beating, and generational IPV were associated with elevated odds of child abuse. Findings indicate possible high levels of unmet child protection needs, and stress the need for professionals working with children to employ culturally-sensitive methods in investigating social determinants of child abuse.

## Introduction

Globally, child abuse (or child maltreatment) is a significant public health problem extending beyond culture, social context and race. ^1^ Child abuse consists of any acts of commission or omission by a parent, caregiver or other adult resulting in harm, potential for, or threat of harm to a child (0-18 years of age) even if the harm is unintentional. ^2^ The World Health Organization (WHO) estimates that 40 million children aged 0-14 years globally suffer from abuse and neglect that require health and social care. ^3^ The extent and trend of national or global rates, ^4^ and determinants of child abuse are largely unknown. Studies in Egypt are sparse, estimating that 37% of children in Egypt suffer physical punishment with varying degrees of severity; 5 these acts of punishment, presumably committed as acts of child discipline, are engendered by a culture that places a high premium on child obedience and the positive effects of discipline.^6,7^

Social determinants of health (SDH) are conditions in which people are born, grow, live, work and age, including the health system.^8^ These conditions provide the freedom people need to live lives they value,^9^ and are shaped by the distribution of money, power and resources at global, national and local levels. SDH that perpetuate child abuse can be avoided by reasonable societal level action; however, that they are not avoided indicates that they are unfair, unnecessary, unjust, and therefore inequitable.^10^ Given children’s need for safe, healthy, nurturing, and responsive living environments, the SDH that perpetuate child abuse are numerous, and need to be examined to understand the association between child abuse and intimate partner violence (IPV). 

Children exposed to child abuse are often exposed to co-occurring domestic violence (DV) and environmental stressors.^11^ Households frequently experiencing IPV are commonly poor, undergo marital problems, life stressors, and other negative aspects of family life, including low parental education, unemployment, insufficient income, and substance abuse.^12, 13^ Other factors associated with increased risk for child abuse include young child age, minority status, and parental stress, ^14^ immigrant families, single-parent families, stepfamilies, families with three or more children, children 0 - 3 years old,^15, 16^ female sex, and older adolescence. Perpetrator-related risk factors such as parental mental health, chronic illness, criminal history, alcohol or drug abuse, and parental skills have also been implicated with child abuse and IPV.^11, 17, 18^

Knowledge of how the social determinants of child abuse operate and interact is an important first step towards developing interventions and policy-level change needed to improve the lives of affected children and families. To assess for associations, the following hypotheses were tested: 

**Hypothesis I:** The risk of experiencing child abuse will be higher for children exposed to domestic violence, even after controlling for potential confounders;

**Hypothesis II:** Mothers with tolerant attitudes towards wife beating will be more likely to abuse their child than those who do not tolerate wife beating;

**Hypothesis III:** Women exposed to generational IPV i.e. who had witnessed domestic violence in childhood, will be more likely to perpetrating child abuse, compared to those who were not so exposed; and

**Hypothesis IV:** Children in families of higher socio-economic position (SEP), as indicated by educational level of respondent or partner, and household wealth index, will be at lower risk of experiencing abuse compared to those of lower SEP. 

The aim of this study was two-fold: i) to determine the prevalence of child abuse in Egypt; and ii) to investigate factors associated with maternal abuse as social determinants of child abuse.

## Methods

The 2005 Egyptian Demographic and Health Survey (2005 EDHS) conducted between February and July 2005 was used for this study.^19^ This nationally-representative household survey aimed at providing information about reproductive health and socio-economic characteristics was conducted by face-to-face interviews using a standard questionnaire in Arabic language.^20^ We focused primarily on acts of child abuse by mothers, their exposure to IPV, and their attitude towards wife beating, as data on male adults in the household were not collected . Data on IPV was collected in accordance with the WHO’s ethical and safety recommendations for research on domestic violence,^21^ which ensures women’s safety, maximizes disclosure of actual violence, ensures that informed consent is obtained, and guarantees the privacy of respondents. 

Data was collected by multistage sampling; the first sampling stage selected 682 primary sampling units (PSU) (289 shiakhas/towns and 393 villages) from a list of shiakhas/towns and villages in each governorate (Urban Governorates, urban Lower Egypt, rural Lower Egypt, urban Upper Egypt, rural Upper Egypt and the Frontier Governorates). The second sampling stage selected two segments from each PSU, a household listing obtained from each segment. Using the household lists, a systematic sample of 22, 807 households was selected for interview. All ever-married women 15-49 present in the sampled households on the night before the interview were eligible for inclusion in the survey. A sub-sample of one-third of all households in each segment was selected for the anemia-testing component of the survey; one woman in each household in this sub-sample was selected for questions about domestic violence. From the selected households, a total of 19, 565 eligible women aged 15 - 49 years were interviewed, resulting in a 99.5% response rate. Of this number, 19, 474 women were selected from the households, and these had 17,552 children at the time of the interviews. 

The primary outcome variable was acts of child abuse by mothers, which were measured using responses to questions asked to the mothers about whether they had ever carried out the following acts of abuse against their child: i) shouting at their children; ii) striking their children; and iii) or slapping their children. Responses were in the form of dichotomous ‘yes’ or ‘no’ variables. 

The main exposures included: (1) Exposure to IPV, assessed using the Conflict Tactics Scale,^22^ and defined as any act of physical, emotional, or sexual abuse by a current or former husband or intimate partner, whether cohabiting or not. ^23^ A composite ‘yes’ or ‘no’ variable “any IPV” was created from responses to 11 questions asked respondents about ever experiencing one or several of the acts of abuse by a current or former husband or intimate partner namely: i) pushing, shaking or throwing something at her; ii) slapping her or twisting her arm; iii) punching or hitting her with something harmful; iv) kicking or dragging her; v) strangling or burning her; vi) threatening her with a weapon (e.g. gun or knife); vii) attacking her with a weapon; viii) humiliating her in public; ix) threatening her or someone close to her; and x) forced sexual intercourse, where “any IPV” was defined as exposure to one or several of the experiences perpetrated by a husband/partner ever. Reliability of “any IPV”, indicated by Cronbach’s alpha (α) was .798. (2) Respondent justifies wife beating, a composite ‘yes’ or ‘no’ variable created from responses to five questions enquiring whether respondents justify abuse of a woman for such reasons as: when she goes out without telling him, neglects the children, argues with him, refuses to have sex with him, and burns the food. ‘Yes’ was defined as the women’s responses of ‘yes’ to one or several of these attitude questions, and ‘no’ as responses of ‘no’ to all the attitude questions. Cronbach’s alpha (α) was .907. (3) History of generational IPV, a composite binary ‘yes’ or ‘no’ variable and created from responses to the questions “ever physically hurt by: mother” and “ever physically hurt by: father”; Cronbach’s alpha (α) was .68. 

Confounding variables included: (1) Demographic variables, including: i) Respondent’s age (15-19, 20-24, 25-29, 30-34, 35-39, 40-44, and 45-49); ii) Marital status (formerly married, and currently married ); iii) Sex of the child, (female, and male); iv) Age of child (years) (0-4, 5-9, 10-14, and ≥ 15; and v) Number of children in family (≤ 2, 3-5, and ≥ 6). (2) Socio-economic variables, including: i) Respondent’s educational level (no education, primary, and secondary or higher); ii) Partner’s educational level (no education, primary, and secondary or higher); iii) Respondent’s current working status, a measure of economic empowerment (not working, and working); iv) Partner’s current working status (not working, and working); v) Household wealth index, a measure of respondents’ economic status,^24^ using principal component analysis to derive wealth index factor scores that are then divided into five percentiles (from the poorest 20% to the richest 20%); vi) Who decides how to spend money, an indicator of financial autonomy (respondent alone, respondent and husband/partner, and husband/partner and other); and vii) Type of earnings, an indicator of possible financial stress (not paid, cash and kind, in kind only, and cash only). (3) Geographical variables, including: i) Place of residence (urban, and rural).

To determine the social determinants of child abuse associated with maternal exposure to IPV, cross tabulations were performed to examine differences in the distribution of the outcome, exposure, and confounding variables. Bivariate logistic regression models included the confounding variables all entered in a single block to control for possible confounding between these variables. Multivariate analyses using a series of logistic regression models were run iteratively using stepwise regression (backward method), with the variable having the least level of significance being removed at each step until only significant variables remained since the goal of this study was to derive a model with the best fit.^25^ By step 10 of the “any abuse” model, several variables had been dropped during the modeling process, and their subsequent reintroduction did not significantly affect other variables and, thus, they were removed from the model. Alternative analyses were performed with the “Enter” command i.e. entering all the variables in a block; this resulted in all the dropped variables being non-significant, therefore validating the making the backward method of stepwise regression and the retained variables as the best model fit. In the acts of child abuse models, variables not in the final model were dropped earlier in the modeling process, reintroduced, and were found to remain non-significant without changing other predictors; hence they were not retained in the final models. The direction and magnitude of associations were expressed as odds ratios (ORs) and their 95 percent confident interval levels (95% CI), with the analyses conducted using Predictive Analytics Software (PASW) version 18.

## Results

Of the 14,016 women in the study, 91% (n=12694) reported shouting, 69% (n=9684) striking, and 39% (n=5515) slapping their child/ children when they did mistakes during the 12 months prior to the study. The majority (n=9439, 54%) of children were male, aged 9 years or younger (n=13698, 78%), and were 5 or fewer in the family (n=17,572, 90%). Most women were 25 - 29 years old (n=3780, 20%), and resident in urban areas (n=11379, 58%). Other socio-demographic characteristics are shown in Table 1.

**Table 1:** Socio-demographic characteristics of the study population.

Characteristics	N (%)
**Sex of child**	
Male	9439 (54)
Female	8113 (46)
**Total**	**17552 (100)**
**Age of child (years)**	
0 - 4	9894 (56)
5 - 9	3804 (22)
10 - 14	2176 (12)
≥ 15	1678 (10)
**Total**	**17552**
**Number of children in family**	
≤ 2	9052 (46)
3 - 5	8520 (44)
≥ 6	1902 (10)
**Total**	**19474**
**Women’s age (groups)**	
15 - 19	858 (4)
20 - 24	3008 (15)
25 - 29	3780 (20)
30 - 34	3189 (16)
35 - 39	3186 (16)
40 - 44	2827 (15)
45 - 49	2626 (14)
**Total**	**19474**
**Respondent’s educational level**	
No education	6934 (35)
Primary	3064 (16)
Secondary or higher	9476 (49)
**Total**	**19474**
**Partner’s educational level**	
No education	4603 (24)
Primary	3829 (20)
Secondary or higher	11008 (56)
**Total**	**19440**
**Marital status **	
Formerly married	1340 (7)
Currently married	18134 (93)
**Total**	**19474**
**Respondent’s Current working status**	
Not working	15243 (78)
Working	4192 (22)
**Total**	**19435**
**Partner’s current working status**	
Not working	15180 (78)
Working	4294 (22)
**Total**	**19474**
**Wealth index**	
Poorest	4227 (22)
Poorer	3882 (20)
Middle	3669 (19)
Richer	3791 (19)
Richest	3905 (20)
**Total**	**19474**
**Place of residence **	
Rural	11379 (58)
Urban	8095 (42)
**Total**	**19474**
**Who decides how to spend money**	
Respondent alone	712 (25)
Respondent and husband/partner	1938 (69)
Husband/partner and Other	174 (6)
**Total**	**2824**
**Type of earnings**	
Not paid	867 (20)
Cash and kind	167 (4)
In kind only	116 (3)
Cash only	3151 (73)
**Total**	**4301**

N = Total number; % = Percentage

The vast majority of women who had experienced IPV shouted on (n=1401, 93%), and struck (n=1153, 77%) their child. Similarly, a majority of the women justified wife beating and experienced generational IPV (see Table 2). Among women who abused their children, the proportion of currently married women was generally higher than that of formerly married women. Majority of abused children were ≤4 years, and living ≤5 per household. Other socio-demographic characteristics are shown in Table 3.

**Table 2:** Proportion of children within each category of abuse by factors related to abuse among the women in the study.

Characteristics	Shouted at children			Struck children			Slapped children		
	No	Yes		No	Yes		No	Yes	
	N (%)	N (%)	Total	N (%)	N (%)	Total	N (%)	N (%)	Total
Intimate partner violence			****			****			****
No	283 (11)	2362 (89)	**2645**	946 (36)	1700 (64)	**2646**	1741 (66)	904 (34)	**2645**
Yes	100 (7)	1401 (93)	**1501**	349 (23)	1153 (77)	**1502**	793 (53)	707 (47)	**1500**
Justifies wife beating			****			****			****
No	700 (11)	5898 (89)	**6598**	2378 (36)	4221 (64)	**6599**	4453 (68)	2136 (32)	**6589**
Yes	620 (8)	6757 (92)	**7377**	1944 (26)	5434 (74)	**7378**	4024 (55)	3351 (45)	**7375**
Generational IPV			****			****			****
No	323 (10)	2966 (90)	**3289**	1092 (33)	2199 (67)	**3291**	2038 (62)	1251 (38)	**3289**
Yes	61 (7)	792 (93)	**853**	200 (23)	653 (77)	**853**	492 (58)	360 (42)	**852**

P-value: *** p<0.001; **p<0.01; *p<0.05; ns = not significant

**Table 3:** Proportion of children within each category of abuse by characteristics of women in the study.

Characteristics	Shouted at children			Struck children			Slapped children		
	No	Yes		No	Yes		No	Yes	
	N (%)	N (%)	Total	N (%)	N (%)	Total	N (%)	N (%)	Total
Women’s age (groups)		***	****		***	****		***	****
15 - 19	3 (11)	25 (89)	**28**	4 (14)	24 (86)	**28**	16 (57)	12 (43)	**28**
20 - 24	47 (5)	960 (95)	**1007**	163 (16)	844 (84)	**1007**	485 (48)	522 (52)	**1007**
25 - 29	154 (6)	2571 (94)	**2725**	494 (18)	2231 (82)	**2725**	1391 (51)	1331 (49)	**2722**
30 - 34	172 (6)	2679 (94)	**2851**	609 (21)	2242 (79)	**2851**	1534 (54)	1315 (46)	**2849**
35 - 39	234 (8)	2737 (92)	**2971**	886 (30)	2086 (70)	**2972**	1823 (61)	1143 (39)	**2966**
40 - 44	329 (13)	2156 (87)	**2485**	1086 (44)	1399 (56)	**2485**	1742 (70)	743 (30)	**2485**
45 - 49	383 (20)	1565 (80)	**1948**	1090 (56)	858 (44)	**1948**	1503 (77)	443 (23)	**1946**
Sex of child		**	****		ns	****		*	****
Male	666 (9)	6859 (91)	**7525**	2284 (30)	5243 (70)	**7527**	4498 (60)	3021 (40)	**7519**
Female	655 (10)	5834 (90)	**6489**	2048 (32)	4441 (68)	**6489**	3996 (62)	2488 (38)	**6484**
Respondent’s educational level		ns	****		***	****		***	****
No education	508 (10)	4792 (90)	**5300**	1488 (28)	3813 (72)	**5301**	2898 (55)	2396 (45)	**5294**
Primary	240 (10)	2155 (90)	**2395**	783 (33)	1612 (67)	**2395**	1425 (59)	969 (41)	**2394**
Secondary or higher	573 (9)	5746 (91)	**6319**	2061 (33)	4259 (67)	**6320**	4171 (66)	2144 (34)	**6315**
Partner’s educational level		*	****		***	****		***	****
No education	367 (10)	3128 (90)	**3495**	1063 (30)	2433 (70)	**3496**	1951 (56)	1540 (44)	**3491**
Primary	256 (9)	2707 (91)	**2963**	824 (28)	2139 (72)	**2963**	1673 (56)	1287 (44)	**2960**
Secondary or higher	694 (9)	6834 (91)	**7528**	2439 (32)	5090 (68)	**7529**	4858 (65)	2666 (35)	**7524**
Marital status		***	****		***	****		***	****
Formerly married	150 (18)	694 (5)	**844**	400 (47)	444 (53)	**844**	605 (71)	238 (29)	**843**
Currently married	1171 (9)	11999 (91)	**13170**	3932 (30)	9240 (70)	**13172**	7889 (60)	5271 (40)	**13160**
Current working status		**	****		***	****		***	****
Not working	994 (9)	9874 (91)	**10868**	3123 (29)	7746 (71)	**10869**	6399 (59)	4459 (41)	**10858**
Working	327 (10)	2818 (90)	**3145**	1209 (38)	1937 (62)	**3146**	2094 (67)	1050 (33)	**3144**
Partner’s current working status		ns	****		***	****		***	****
Not working	977 (9)	9643 (91)	**10620**	3060 (29)	7561 (71)	**10621**	6272 (51)	4338 (49)	**10610**
Working	344 (10)	3050 (90)	**3394**	1272 (37)	2123 (63)	**3395**	2222 (65)	1171 (35)	**3393**
Wealth index		***	****		***	****		***	****
Poorest	304 (10)	2855 (22)	**3159**	746 (24)	2414 (76)	**3160**	1572 (50)	1585 (50)	**3157**
Poorer	238 (8)	2595 (92)	**2833**	760 (27)	2073 (73)	**2833**	1553 (55)	1278 (45)	**2831**
Middle	228 (9)	2355 (91)	**2583**	748 (30)	1835 (70)	**2583**	1553 (60)	1026 (40)	**2579**
Richer	226 (9)	2410 (91)	**2636**	816 (31)	1820 (69)	**2636**	1662 (63)	971 (37)	**2633**
Richest	325 (12)	2478 (88)	**2803**	1262 (45)	1542 (55)	**2804**	2154 (77)	649 (28)	**2803**
Place of residence		ns	****		***	****		***	****
Rural	539 (9)	5303 (91)	**5842**	2046 (47)	3797 (39)	**5843**	3834 (66)	2005 (34)	**5839**
Urban	782 (9)	7390 (91)	**8172**	2286 (28)	5887 (72)	**8173**	4660 (57)	3504 (43)	**8164**
Age of child (years)		***	****		***	****		***	****
0 - 4	398 (5)	6788 (95)	**7186**	1401 (19)	5785 (81)	**7186**	3720 (52)	3460 (48)	**7180**
5 - 9	305 (8)	3330 (92)	**3635**	1081 (30)	2555 (70)	**3636**	2251 (62)	1383 (38)	**3634**
10 - 14	333 (17)	1689 (83)	**2022**	1070 (53)	953 (47)	**2023**	1563 (77)	455 (23)	**2018**
≥ 15	244 (29)	603 (71)	**847**	649 (77)	198 (23)	**847**	744 (88)	103 (12)	**847**
Number of children in family		***	****		**	****		ns	****
≤ 2	363 (9)	3773 (91)	**4136**	1224 (28)	2912 (30)	**4136**	2536 (61)	1597 (39)	**4133**
3 - 5	726 (9)	7294 (91)	**8020**	2482 (57)	5540 (57)	**8022**	4864 (61)	3149 (39)	**8013**
≥ 6	232 (12)	1626 (88)	**1858**	626 (15)	1232 (13)	**1858**	1094 (59)	763 (41)	**1857**
Who decides how to spend money		*	****		*	****		***	****
Respondent alone	49 (9)	503 (91)	**552**	11 (3)	341 (97)	**352**	211 (52)	194 (48)	**405**
Respondent & husband/partner	176 (11)	1397 (89)	**1573**	668 (42)	905 (58)	**1573**	668 (61)	420 (39)	**1088**
Husband/partner and Other	7 (5)	125 (95)	**132**	42 (32)	90 (68)	**132**	42 (42)	59 (58)	**101**
Type of earnings		*	****		***	****		***	****
Not paid	50 (7)	640 (93)	**690**	166 (24)	525 (76)	**691**	348 (16)	342 (29)	**690**
Cash and kind	12 (8)	131 (92)	**143**	41 (29)	102 (71)	**143**	78 (3)	65 (6)	**143**
In kind only	9 (9)	88 (91)	**97**	22 (23)	74 (77)	**96**	38 (2)	59 (5)	**97**
Cash only	274 (11)	2198 (89)	**2472**	1049 (42)	1423 (58)	**2472**	1765 (79)	706 (60)	**2471**

P-value: *** p<0.001; **p<0.01; *p<0.05; ns = not significant

In the multivariate logistic regression analyses, adjusting for potential confounders between child abuse and factors associated with maternal abuse, experience of IPV remained associated with elevated odds of mothers striking [adjusted odds ratio (AOR) = 1.57, 95% CI = 1.03 - 2.40; P = 0.035] and slapping [AOR = 1.57, 95% CI = 1.03 - 2.38; P = 0.034] their child compared to mothers with no experience of IPV. Women justifying wife beating was associated with higher odds of shouting at a child [AOR = 2.32, 95% CI = 1.02 - 5.28; P = 0.045], whilst generational IPV was associated with higher odds of shouting at [AOR = 2.95, 95% CI = 1.08 - 8.05; P = 0.034] and striking a child [AOR = 1.73, 95% CI = 1.09 - 2.75; P = 0.020], compared to not justifying wife beating and lack of generational IPV, respectively. The odds of women slapping their child decreased with increasing age; the highest odds were observed among women aged 30 - 34 years. In addition, women aged 20 - 24, 25 - 29, and 30 - 34 years were also at higher odds of striking their child compared to women aged 45 - 49 years. Female children [AOR = 0.57, 95% CI = 0.40 - 0.82; P = 0.002] were at lower odds of being slapped by their mother than male children.

The odds of a mother slapping her child was highest among women in the poorest wealth quintile [AOR = 3.12, 95% CI = 1.27 - 7.65; P = 0.013] compared to the richest quintile; the odds decreased as the wealth quintile increased. Likewise, women in the poorer [AOR = 2.81, 95% CI = 1.20 - 6.59; P = 0.018] and richer wealth quintiles [AOR = 1.71, 95% CI = 1.09 - 2.69; P = 0.019] were at higher odds of striking their child compared to women in the richest quintile. Compared to children 15 years or older, children aged 0 - 4 years had the highest odds of experiencing all the forms of child abuse; these odds became lower the older (5 - 9 years) the child. With the exception of children who were slapped by their mother, households with 3 - 5 and 6 or more children were at higher odds of experiencing any abuse than households with ≤2 children. In contrast, women who decided along with their husband/partner how to spend money [AOR = 0.48, 95% CI = 0.32 - 0.72; P< 0.001] were at lower odds of slapping their child compared to those women who decided alone (Table 4).

**Table 4:** Adjusted Odds Ratio (OR) for the social determinants of abuse against children in Egypt.

Characteristics	Shouted at children OR (95% CI)	Struck children OR (95% CI)	Slapped children OR (95% CI)
**Intimate partner violence (any IPV)**			
No	1	1	1
Yes	1.76 (0.79 - 3.92)	1.57 (1.03 - 2.40)	1.57 (1.03 - 2.38)
**Respondent justifies wife beating**			
No	1	1	1
Yes	2.32 (1.02 - 5.28)	1.11 (0.73 - 1.69)	0.90 (0.58 - 1.39)
**Generational IPV**			
No	1	1	1
Yes	2.95 (1.08 - 8.05)	1.73 (1.09 - 2.75)	1.41 (0.91 - 2.18)
**Respondents’ age (groups)**			
15 - 19	‡	1.68 (0.38 - 7.35)	‡
20 - 24	1.84 (0.44 - 7.71)	3.94 (1.65 - 9.39)	2.95 (0.68 - 12.71)
25 - 29	2.45 (0.71 - 7.07)	2.70 (1.38 - 5.28)	2.54 (0.99 - 6.46)
30 - 34	1.82 (0.71 - 4.62)	2.84 (1.56 - 5.18)	3.35 (1.49 - 7.51)
35 - 39	1.53 (0.71 - 3.29)	1.58 (0.92 - 2.71)	2.80 (1.32 - 5.94)
40 - 44	0.77 (0.44 - 1.34)	0.90 (0.64 - 1.26)	2.10 (1.01 - 4.37)
45 - 49	1	1	1
**Marital status**	‡	‡	‡
Formerly married			
Currently married			
**Sex of child**			
Female	0.77 (0.44 - 1.34)	0.90 (0.64 - 1.26)	0.57 (0.40 - 0.82)
Male	1	1	1
**Respondent’s educational level**			
No education	1.50 (0.34 - 6.69)	1.05 (0.41 - 2.35)	0.97 (0.41 - 2.02)
Primary	2.95 (0.45 - 19.35)	0.52 (0.21 - 1.30)	1.29 (0.54 - 3.05
Secondary or higher	1	1	1
**Partner’s educational level**			
No education	0.73 (0.18 - 3.01)	1.36 (0.62 - 2.99)	1.59 (0.78 -3.27)
Primary	1.38 (0.34 - 5.59)	1.50 (0.69 - 3.25)	1.08 (0.54 - 2.15)
Secondary or higher	1	1	1
**Respondent’s current working status**			
Not working	0.44 (0.07 - 2.74)	1.23 (0.32 - 4.75)	0.59 (0.18 - 1.91)
Working	1	1	1
**Partner’s current working status**	‡		
Not working		0.36 (0.02 - 7.19)	1.84 (0.08 - 43.60)
Working		1	1
**Wealth index**			
Poorest	0.42 (0.09 - 1.98)	2.05 (0.79 - 5.31)	3.12 (1.27 - 7.65)
Poorer	1.09 (0.24 - 4.99)	2.81 (1.20 - 6.59)	2.58 (1.17 - 5.70)
Middle	1.67 (0.51 - 5.41)	1.37 (0.74 - 2.55)	1.84 (0.96 - 3.50)
Richer	0.78 (0.37 - 1.64)	1.71 (1.09 - 2.69)	1.80 (1.10 - 2.94)
Richest	1	1	1
**Place of residence **			
Rural	0.49 (0.23 - 1.04)	0.80 (0.51 - 1.26)	0.76 (0.48 - 1.20)
Urban	1	1	1
**Age of child (years)**			
0 - 4	5.84 (1.82 - 18.72)	4.71 (1.96 - 11.32)	4.70 (1.45 - 15.25)
5 - 9	2.97 (1.16 - 7.58)	3.39 (1.49 - 7.71)	2.46 (0.78 - 7.76)
10 - 14	2.13 (0.84 - 5.37)	1.26 (0.53 - 2.99)	1.07 (0.31 - 3.64)
≥ 15	1	1	1
**Number of children in family**			
≤ 2	1	1	1
3 - 5	2.56 (1.33 - 4.95)	1.67 (1.10 - 2.53)	1.25 (0.80 - 1.96)
≥ 6	1.07 (0.25 - 4.61)	2.81 (1.09 - 7.22)	2.14 (0.87 - 5.24)
**Who decides how to spend money**			
Respondent alone	1	1	1
Respondent and husband/partner	0.85 (0.41 - 1.59)	0.86 (0.57 - 1.30)	0.48 (0.32 - 0.72)
Husband/partner and other	1.95 (0.82 - 4.67)	1.27 (0.52 - 3.08)	0.63 (0.27 - 1.46)
**Type of earnings**			
Not paid	3.70 (0.39 - 34.95)	1.89 (0.64 - 5.57)	1.61 (0.67 - 3.91)
Cash and kind	1.44 (0.75 - 2.77)	1.44 (0.75 - 2.77)	1.87 (0.88 - 3.96)
In kind only	1.20 (0.58 - 2.51)	1.20 (0.58 - 2.51)	1.20 (0.58 - 2.51)
Cash only	1	1	1

‡ Data not computed due to small number 1= reference category

## Discussion

This study found that child abuse is relatively common in Egypt. Majority of the children had experienced some form of abusive act, and the least frequent act of abuse was slapping. These rates are comparable to those reported in other studies conducted in Egypt,^7,8,26^ and elsewhere,^27^ but are higher than those 4 - 36% among mothers in Chile, Egypt, India and the Philippines.^28^

The finding that mothers who experienced IPV were more likely to be abusive towards their children corroborates findings from other studies, ^11, 29^ suggesting an abused mother’s inability to respond effectively to child misbehaviour due to the stress and psychological impact of IPV victimization,^30^ or an abused mother’s effort to avoid a misbehaving child angering an abusive partner, thus increasing the risk of both the woman and her abusive male partners,^31^ perpetrating child abuse. This finding provides support for our first hypothesis that the risk of experiencing child abuse, even after controlling for potential confounders. IPV exposure is reported to be the most potent factor for predicting parental abuse of a child;^7^ this carries important implications for child abuse prevention efforts, which need to directly address existing IPV within families. However, not everyone agrees, as some studies suggest that women living with domestic abuse are no more likely than other women to abuse their children.^32^ Support for our second hypothesis, that mothers with tolerant attitudes towards wife beating will be more likely to abuse their child, could be found in the results that women who justified wife beating were more likely to shout at their child. As far as the authours are aware, this is the first study with this finding, consistent with those previously reported,^26^ as well as those reported in relation to corporal punishment. ^33,34^ In Egypt, violent behaviour, including stern acts of discipline against children, is commonplace and a widely accepted cultural practice in contrast to high income countries. There is therefore a need to break the “invisibility” and social acceptance of child abuse by advocating for policies, laws, and services for prevention and response, in addition to educational campaigns aimed at changing social norms and individual attitudes that are harmful to children. 

The association between exposure to generational IPV and child abuse (shouting at, and striking a child) is consistent with findings from studies that found that physically abused parents themselves had about 2.6 - 5 times higher risk of being physically abusive to their children.^35, 36^ In addition to validating our third hypothesis of that women’s exposure to generational will be more likely to perpetrating child abuse, this finding appears to support a social learning approach to understanding the cycle of violence,^37^ whereby the respondents may have learned and justified violent behaviour by directly witnessing or experiencing parental abuse. ^38^ Although about one-third of abused individuals are reported to go on to abuse their children, ^37^ it is noteworthy that most abused people do not necessarily become abusive. 

The finding that younger women were more likely to strike and slap their children than older women endorses other studies from elsewhere; ^14^ this may be linked to other factors, such as lower economic status, lack of social support, and higher stress levels compared to older mothers. We also found that female children were less likely to be slapped by their mother, corroborating findings from other studies.^34, 39^ Explanations for boys’ increased vulnerability to physical abuse (slapping) is unclear; however such punishments may be seen as a preparation for future adult roles and responsibilities and the considered need more stricter physical discipline.^40^ Wide cultural differences in norms between societies with respect to the role of women and the values attached to male and female children could account for a large part of these differences. The finding that living in households with poorer wealth quintile increased the likelihood of a child being struck and slapped is concordant with other studies, ^6, 41^ but contradicting others where no association was observed.^42^ Whilst poorer people do not all abuse their children, poverty tends to contribute to negative patterns of family functioning by interacting with other risk factors such as depression, substance abuse, and social isolation to increase the likelihood of abuse. In addition, this finding lends support to our fourth hypothesis that children in families of higher SEP will be at lower risk of experiencing abuse compared to those of lower SEP, although neither respondents’ or their partners’ educational level were non-significantly associated with the different acts of child abuse.

Our finding that younger children (0 - 4, and 5 - 9 years), compared to older children ≥ 15 years were more likely to experience all the forms of child abuse is in agreement with other studies,^15, 16^ that attribute higher risks for families with children less than three years old. As children are not responsible for being victims of abuse, their increased vulnerability is plausibly dependent on interactions between their small physical size, early development, constant need for care, and parental characteristics such as stress etc. We also found that families with 3 - 5 children were more likely to be abusive (except slapping) towards their children than parents with fewer children, supporting findings from other studies. ^15, 16^ Plausible explanations include household overcrowding,^6^ or unstable family environments with frequently changing (moving in and out) household composition. The finding that women who had joint financial autonomy i.e. partook jointly with their husband/partner on how money was spent in the home were less likely than those who took decisions alone to abuse (slap) their child is a unique finding that needs further investigating. Plausible explanations could be that households in which women have joint financial autonomy are more egalitarian, providing children with more stable and stress-free environments with lower likelihood of abuse. This egalitarian relationship encourages couples to settle household disputes by negotiation, ^43^ rather than violence that could “spill over” to the children. In contrast, households where women had sole financial autonomy could appear more threatening to more traditional men that propagate IPV,^44^ thereby increasing the likelihood of child abuse.

Our findings suggest a number of practice and policy implications. First, the possibility of a significant gap between the numbers of children with child protection needs may indicate a high level of unmet needs. Second, professionals at child services need to employ culturally-sensitive methods in investigating child abuse in order to facilitate timely identification and response to child abuse cases. Third, there is a need to enlighten parents about methods of positive corrective treatment of their children, which may necessitate change in societal norms of child disciplinary methods. The findings of this study should however be interpreted with caution, given that a complete picture of child abuse and neglect may not have emerged due to inability to study male adults in the household as a result of lack of data. This study has several limitations. Firstly, estimating the true prevalence rates for child abuse may require interviewing both the perpetrators and the victims since child abuse often occurs within the privacy of home or in private settings where detection and disclosure are more difficult. Secondly, data was based on respondents’ self-report of abusive acts without accounting for fathers’ or other caregivers’ use of abusive acts or independent verification of these. Thirdly, the cross-sectional nature of the study precludes the drawing of causal inference between child abuse and IPV; prospective longitudinal studies would be required to disentangle the temporal ordering of abuse and determinants. Strengths include the low- and middle-income context of the study, the nationally-representative data, and the novel contribution of the findings. 

In summary, experience of IPV, women justifying wife beating, and generational IPV were associated with elevated odds of child abuse. Findings indicate possible high levels of unmet child protection needs; the need for culturally-sensitive methods in investigating child abuse by professionals working with children and young people so as to facilitate the timely identification and response to child abuse cases.

## Acknowledgements

The author is grateful to Opinion Research Corporation Macro International, Incorporated, (ORC Macro Inc.), Calverton, USA for the data used in this study.
